# Orsay virus variants isolated from wild *Caenorhabditis elegans* nematodes, France

**DOI:** 10.1128/mra.00550-25

**Published:** 2025-09-10

**Authors:** Chika Fujii, Susan Vernon, Marie-Anne Félix, David Wang

**Affiliations:** 1 Department of Molecular Microbiology, School of Medicine, Washington University in St. Louis 12275 , St. Louis, Missouri, USA; 2 Institute of Biology of the Ecole Normale Supérieure (IBENS), Paris, France; 3 Department of Pathology & Immunology, School of Medicine, Washington University in St. Louis 12275, St. Louis, Missouri, USA; Katholieke Universiteit Leuven, Leuven, Belgium

**Keywords:** Orsay virus, *Caenorhabditis elegans*

## Abstract

Four new variants of Orsay virus were identified from wild isolates of *Caenorhabditis elegans* nematodes collected from decaying plant matter in France. Near-complete genomes of the viruses were determined by metagenomic sequencing. The four genomes share 96.1–98.9% nucleotide identity with the reference Orsay virus sequence JUv1580.

## ANNOUNCEMENT

**Fig 1:**
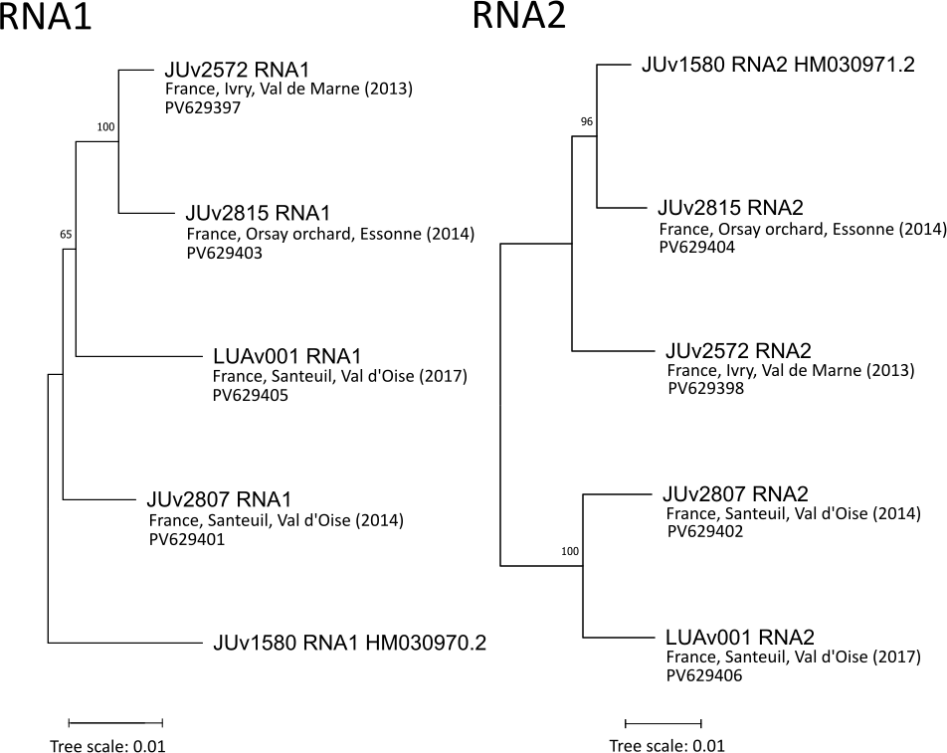
Maximum likelihood phylogenetic trees of the Orsay virus RNA1 and RNA2 segments, constructed with the reference JUv1580 and the four newly identified virus strains. Sequences were aligned with Clustal Omega, and a maximum likelihood phylogenetic tree was constructed with MEGA11 (8) with 1,000 bootstrap replicates. JUv1580 was included in the analysis as the reference genome. The node values represent the probability with which the sequences clustered together in the bootstrap test with 1,000 replicates.

Orsay virus is a single-stranded, positive-sense, and bi-segmented RNA virus that infects *Caenorhabditis elegans* (*C. elegans*) nematodes (1). The virus has yet to be formally classified by the International Committee on Taxonomy of Viruses, but it is closely related to viruses in the *Nodaviridae* family (1). The originally discovered wild isolate JUv1580 has provided molecular biological insights into the Orsay virus life cycle, and its RNA1 (HM030970.2) and RNA2 (HM030971.2) segment sequences have served as a reference genome. RNA1 encodes the RNA-dependent RNA polymerase (RdRp) (2), whereas RNA2 contains two open reading frames encoding the alpha and delta proteins. The alpha protein forms the viral capsid (3), while the delta protein is implicated in viral exit (4). The alpha and delta proteins also exist in a fused form (5). Here, we report the sequences of four new Orsay virus variants, discovered in nematodes dwelling in decaying plant matter in France. The four Orsay virus variants, JUv2572, JUv2807, JUv2815, and LUAv001, were isolated from sites in Ivry (GPS 48.8092, 2.3862), Santeuil (GPS 49.12165, 1.95101), Orsay (GPS 48.7015, 2.1725), and Santeuil (GPS 49.121, 1.951), respectively. Viruses in infected nematodes were maintained as described previously (1) on agar plates seeded with *Escherichia coli* strain OP50. For isolation of RNA, infected nematodes were homogenized in TRIzol (Invitrogen), followed by extraction of the total RNA using the Direct-zol RNA extraction Kit (ZYMO). The TruSeq Stranded Total RNA Kit (Illumina) was used for library preparation. The library was subjected to paired-end sequencing on either the Illumina MiSeq (2×250 bp; JUv2572, JUv2815, LUAv001) or the NovaSeq (2×150 bp; JUv2807) platforms. Raw sequencing reads were processed in the CZID platform (6), a metagenomics analysis pipeline for detecting virus sequences in the NCBI database. Within the CZID platform, quality control was performed with Trimmomatic (adapter trimming) and STAR/Bowtie2 (host read removal), assembly was performed using SPAdes, and the assembled contigs were assessed for matches to NCBI’s nucleotide databases. The *C. elegans* host genome was specified to be subtracted in the CZID metagenomics analysis pipeline. The number of reads, average depth of coverage, percentage of GC within the coding-complete genome, percent identity compared to the reference genome (JUv1580), the total lengths of the genomes, and the percent amino acid identities relative to JUv1580 of RdRp, alpha, delta, and alpha-delta fusion proteins are summarized in Table 1. Clustal Omega (7) was used to determine the percent identities relative to the JUv1580 reference. To visualize the relationships between the RNA1 and RNA2 among the variants, a phylogenetic tree was constructed (Fig. 1). The availability of these virus isolates and their genome sequences will enable experimental studies using different Orsay virus strains, as well as studies of viral evolution.

**TABLE 1:** Summary of the sequencing information and features of the four new Orsay virus strains

Virus	Year collected	Total no. of reads	RNA1					RdRp protein amino acid identity (%)	RNA2					Alpha protein amino acid identity (%)	Delta protein amino acid identity (%)	Alpha-delta fusion protein amino acid identity (%)
			No. of reads mapped to RNA1	Average coverage depth (x)	Coding-complete genome GC content (%)	Nucleotide identity compared to the JUv1580 reference (%)	Total length (bp)		No. of reads mapped to RNA2	Average coverage depth (x)	Coding-complete genome GC content (%)	Nucleotide identity compared to the JUv1580 reference (%)	Total length (bp)			
JUv2572	2013	1,478,516	7,579	483	55.9	96.9	3,401	98.3	7,646	664	52.6	98.1	2,768	98.5	98.8	98.4
JUv2807	2014	38,124,337	113,997	4,869	55.7	96.9	3,417	98.4	343,566	19,411	52.7	96.1	2,573	99.2	96.5	97.8
JUv2815	2014	831,671	12,889	845	55.6	96.6	3,394	98.1	17,526	1,563	52.7	98.9	2,523	99.7	100.0	99.7
LUAv001	2017	3,047,176	9,257	562	55.8	96.3	3,396	98.1	13,354	1,088	52.9	96.5	2,560	98.5	96.0	97.1

## Data Availability

The raw sequencing results of this Orsay virus metagenomic sequencing project have been deposited in SRA under the SRR accession numbers SRR33389215 (JUv2572), SRR33389213 (JUv2807), SRR33389212 (JUv2815), and SRR33389211 (LUAv001). The coding-complete genomic sequences of the Orsay virus variants were deposited in GenBank under the accession numbers PV629397 (JUv2572_RNA1), PV629398 (JUv2572_RNA2), PV629401 (JUv2807_RNA1), PV629402 (JUv2807_RNA2), PV629403 (JUv2815_RNA1), PV629404 (JUv2815_RNA2), PV629405 (LUAv001_RNA1), and PV629406 (LUAv001_RNA2).
